# Genomic interplay between deployment exposures and Gulf War illness in Million Veteran Program participants

**DOI:** 10.1186/s40246-026-00951-w

**Published:** 2026-03-27

**Authors:** Dan Qiu, Brenda Cabrera-Mendoza, Jun He, Lea Steele, Rachel Quaden, Kelly M. Harrington, Sarah T. Ahmed, J. Michael Gaziano, Elizabeth J. Gifford, Mihaela Aslan, Drew A. Helmer, Elizabeth R. Hauser, Renato Polimanti

**Affiliations:** 1 Cooperative Studies Program Clinical Epidemiology Research Center (CSP-CERC), VA Connecticut Healthcare System, West Haven, CT USA; 2 Department of Psychiatry, Yale University School of Medicine, 60 Temple, Suite 7A, New Haven, CT 06510 USA; 3 Veterans Health Research Program, Yudofsky Division of Neuropsychiatry, Department of Psychiatry and Behavioral Sciences, Baylor College of Medicine, Houston, TX USA; 4 Million Veteran Program (MVP) Coordinating Center, VA Boston Healthcare System, Boston, MA USA; 5 Department of Psychiatry, Boston University Chobanian & Avedisian School of Medicine, Boston, MA USA; 6 Center for Innovations in Quality, Effectiveness, and Safety (IQuESt), Michael E. DeBakey VA Medical Center, Houston, TX USA; 7 Department of Medicine, Baylor College of Medicine, Houston, TX USA; 8 Department of Medicine, Mass General Brigham, Harvard Medical School, Boston, MA 02115 USA; 9 Department of Veterans Affairs, VA Cooperative Studies Program Epidemiology Center-Durham, Durham, NC USA; 10 Center for Child and Family Policy, Duke Margolis Center for Health Policy, Duke University Sanford School of Public Policy, Durham, NC USA; 11 Department of Internal Medicine, Yale University School of Medicine, New Haven, CT USA; 12 Department of Biostatistics and Bioinformatics, Duke Molecular Physiology Institute, Duke University, Durham, NC USA; 13 Wu Tsai Institute, Yale University, New Haven, CT USA; 14 Department of Chronic Disease Epidemiology, Yale School of Public Health, New Haven, CT USA; 15 Department of Biomedical Informatics and Data Science, Yale School of Medicine, New Haven, CT USA

**Keywords:** Gulf War illness, Military exposure, Veterans, Gene-environment interaction, Polygenic risk, Epigenetics

## Abstract

Veterans of the 1990–1991 Gulf War (GW) experienced an elevated burden of chronic health conditions, most notably Gulf War Illness (GWI). While the disease etiology remains unclear, it is hypothesized that both genetic susceptibility and deployment exposures contribute to GWI risk. We investigated 6,882 GW-deployed Veterans, assessing genetic and epigenetic interactions with GW exposures (ground combat, insect baits, oil well smoke exposure, pyridostigmine bromide pills, biological/chemical warfare agents, and pesticides). We observed nominal evidence of a genome-wide gene–environment interaction variance component (0.38 ± 0.22) related to biological/chemical warfare-agent exposure and identified three loci showing genome-wide significant interactions (*p* < 5 × 10^− 8^; rs78441512, rs145790544, and rs117997207). Also, type-2 diabetes polygenic risk association with GWI is reduced in Veterans exposed to biological/chemical warfare agents, pesticides, and oil well fire smoke during GW deployment. An exploratory computational drug-repurposing analysis highlighted clebopride, rifampicin, and fisetin as compounds potentially targeting GWI-associated molecular pathways. Our GWI epigenome-wide association study identified five sites showing epigenetic interaction with four GW exposures: ground combat, biological/chemical warfare agents, pyridostigmine bromide pill use, and pesticide use. In conclusion, our study demonstrates that both genetic and epigenetic factors interact with GW military exposures to influence GWI vulnerability, highlighting potential druggable pathways to develop novel therapeutic interventions.

**Supplementary Information:**

The online version contains supplementary material available at 10.1186/s40246-026-00951-w.

## Introduction

Approximately one-third of U.S. veterans deployed during the 1990–1991 Gulf War (GW) are reported to have the chronic multisymptom condition known as Gulf War Illness (GWI) [1]. According to the criteria for severe GWI defined by the Centers for Disease Control (CDC) [2], the core symptomatology includes cognitive/mood dysfunction, musculoskeletal pain, and chronic fatigue. Decades after GW, many affected Veterans continue to struggle with these ongoing health issues [3]. It is hypothesized that the interplay between genetic susceptibility and environmental exposures during deployment contributed to the development of GWI [4]. In particular, GW Veterans were exposed to a unique combination of diverse and potentially harmful factors in theater [1, 3]. These include smoke from over 600 burning oil wells in Kuwait, biological/chemical warfare agents (e.g., nerve agents sarin and cyclosarin), and use of pyridostigmine bromide (PB; used as a nerve agent prophylactic). Service members were also exposed (often at excess levels) to numerous pesticides and insect repellants, including organophosphates, pyrethroids, lindane, and N, N-Diethyl-meta-toluamide (DEET). Combustion byproducts like dioxins and dibenzofurans contributed further to toxic exposures related to GW [1, 4]. Despite decades of investigation, key knowledge gaps persist regarding the biological impact of these exposures.

Investigating gene-environment interactions (G×E) between deployment-related exposures and genetic factors may offer critical mechanistic insights into GWI pathogenesis [5]. Emerging research also suggests that epigenetic mechanisms, particularly DNA methylation, may play a key role in mediating the biological impact of environmental exposures on GWI [6, 7]. DNA methylation is essential for regulating gene expression, maintaining genomic stability, and modulating immune and neurological functions [8]. Environmental toxicants have been shown to induce both hypo- and hypermethylation of specific gene regions, potentially leading to persistent alterations in gene function and disease risk [9, 10]. Given the complex and multifactorial nature of GWI, exploring the interaction between environmental exposures and epigenetic modifications offers a promising avenue to better understand the disease etiology.

To investigate how genetic and environmental factors jointly influence GWI susceptibility, we conducted a genome-wide G×E interaction study of GWI in 6,882 GW Veterans (Table S1), examining multiple GW deployment exposures (i.e., proximity to smoke from oil well fires, participation in ground combat, use of PB pills, exposure to biological/chemical warfare agents, use of skin pesticides, and use of insect baits or no-pest strips; Table S2). To gain additional insight into GWI pathogenesis, we also performed polygenic risk scoring-environment interaction (PRS×E) analysis and drug-repurposing analyses. Finally, we performed an exploratory epigenome-wide association study (EWAS) to assess epigenetic mechanisms by which genetic and environmental factors jointly influence GWI susceptibility.

## Methods

### Study design

The present investigation leveraged genetic, epigenetic, and GWI-related data from Veterans who had served during the 1990–1991 GW era. While the sample investigated in the present study is much larger than the majority of GWI molecular studies, genome-wide and epigenome-wide analyses require even large cohorts to characterize the molecular basis and the genomic interplay with environmental risk factors of complex traits such as GWI. To maximize the discovery power of our study and explore new dynamics contributing to GWI pathogenesis, we designed our study to ensure adequate multiple testing correction when possible and conduct exploratory investigations when the sample size was too limited (Table S3). Specifically, our initial analysis to decompose genome-wide G×E variance of GWI across military exposures applied a nominal significance threshold (*p* < 0.05) as an exploratory screening step to identify exposures showing evidence of genome-wide interaction signal. GW exposures passing this threshold were prioritized for follow-up single-variant interaction testing, where a strict genome-wide significance threshold (*p* < 5 × 10⁻⁸) was applied to identify individual variants underlying the observed genome-wide trend. Similarly, we applied a Bonferroni multiple testing correction (*p* < 0.007) to identify statistically significant interactions between polygenic risk and military exposures in the context of GWI. Then, we considered a nominal significance (*p* < 0.05) to identify gene ontology enrichments underlying polygenic risk interactions and used them as input for a genetically informed drug repurposing analysis that applied false discovery rate correction (FDR q < 0.1) to account for the number of molecular compounds tested. Finally, while we applied an initial suggestive threshold (*p* < 0.01) to our epigenetic association analysis, we tested interactive effects of the CpG sites identified by applying an FDR multiple testing correction (FDR q < 0.1).

### Participants

This study was conducted under U.S. Department of Veterans Affairs (VA) Cooperative Studies Program (CSP) #2006, a project that enrolled VA Million Veteran Program (MVP) participants who had served during the 1990–1991 GW era [11]. MVP cohort details have been previously reported [12, 13]. In the MVP CSP #2006 project, eligible participants were identified using U.S. Department of Defense records [11]. Between June 2018 and March 2019, 109,976 MVP participants who served during GW were invited by mail to complete the “MVP 1990–1991 Gulf War Era Survey”. A total of 45,270 (41%) veterans returned completed surveys [14]. Of these, 11,736 GW deployed Veterans have complete genome-wide data. To ensure sufficient sample size for genetic analysis and avoid population stratification biases, the analyses were limited to GW Veterans of European ancestry (EUR) (*N* = 6,882; Tables S1).

Research involving the MVP data was approved by the VA Central Institutional Review Board (CIRB). CIRB approval code for CSP #2006 was “19–26.” The project CSP #2006 was also approved by VA Research and Development Committees in Durham (North Carolina), Houston (Texas), Boston (Massachusetts), and West Haven (Connecticut).

### GWI case definition

GWI was defined using research criteria for severe cases developed by the CDC and recommended by the Institute of Medicine (IOM) [15]. Accordingly, GWI cases were defined by the presence of one or more severe symptoms, lasting 6 months or longer, in at least 2 of 3 defined symptom domains: fatigue domain (fatigue symptom), musculoskeletal domain (pain in muscles, pain in joints, joint stiffness), and mood-cognition domain (difficulty sleeping, difficulty concentrating or remembering, trouble finding words, feeling moody, feeling anxious, feeling depressed). Although other GWI case definitions exist, the present study utilized CDC criteria, because they showed a significant genetic component [16]. The full algorithm used to operationalize GWI definition using MVP survey data is publicly available via the (https://github.com/VA-Phenomics-Library-CIPHER/Gulf-War-Illness).

### Military exposures

The “MVP 1990–1991 Gulf War Era Survey” includes questions that ask Veterans to report on specific GW experiences and exposures potentially encountered during GW deployment (Table S2) [14]. Specifically, Veterans were asked to report whether, during GW deployment, they were directly involved in ground combat and if they were exposed to smoke from oil well fires, biological/chemical warfare agents, took pyridostigmine bromide pills, used pesticide cream or liquids on the skin, wore uniforms treated with pesticides, or used insect baits/pest strips in their living area [11]. We identified presence or absence of GW deployment experiences and exposure based on Veterans’ responses to these questions. Responses marked as “Not Sure”, left blank, or contradictory (e.g., both “Yes” and “No” selected) were excluded from analysis.

### Genetic data

We utilized individual-level genome-wide genotype data available from the MVP cohort. Genotyping, quality control (QC), and relatedness assessment procedures have been previously described [17]. Genotypes were imputed using the Trans-Omics for Precision Medicine (TOPMed) reference panel [18]. Genetic ancestry was assigned using a random forest clustering algorithm informed by reference panels from the 1000 Genomes Project Phase 3 and the Human Genome Diversity Project, classifying individuals into different ancestry groups [19]. We applied standard QC filters to the genetic data, excluding individuals with > 10% missing genotypes and variants with imputation quality (R²) < 0.8, minor allele frequency (MAF) < 1%, call rate < 90%, or deviations from Hardy-Weinberg equilibrium (*P* < 1 × 10⁻²⁰).

As training datasets for the polygenic risk score (PRS) analysis, we used genome-wide association statistics previously generated from EUR cohorts not including MVP (Table S4). These were selected based on previously observed PRS associations with GWI [16]. Specifically, we investigated PRS related to 12 traits: anxiety (meta-analysis between Pan-UK Biobank phecode 300 and FinnGen endpoint KRA_PSY_ANXIETY) [20, 21], chronic pain [22], C-reactive protein [23], depression [24], eosinophil count (Pan UKB Field ID: 30150) [21], irritable bowel syndrome [25], lymphocyte count (Pan UKB Field ID: 30120) [21], neuroticism [26], neutrophil count (Pan UKB Field ID: 30140) [21], posttraumatic stress disorder (PTSD) [27], type-2 diabetes (T2D) [28], and white blood cell count (Pan UKB Field ID: 30000) [21].

### Epigenetic data

DNA methylation data were derived from whole-blood samples collected at the time of enrollment in the MVP cohort and generated using the Illumina Infinium™ Methylation EPIC BeadChip array (version 1), which assays over 850,000 CpG sites across the genome. QC was conducted using the following metrics: at the probe level, CpG sites were excluded if they failed the pOOBAH detection p-value threshold (> 0.05) in ≥ 10% of samples. Samples were retained if at least 96% of probes passed this threshold [29, 30]. After QC, the MVP epigenetic dataset included about 750,000 high-quality CpG sites and 435 GW deployed Veterans.

### Data analysis

#### G×E interaction

We used GCTA software to estimate the proportion of liability variance explained by genome-wide G×E interaction for GWI using genomic restricted maximum likelihood (GREML) analysis [31]. Based on a previous report [32], we considered 28.4% as GWI prevalence in the GREML model. Specifically, univariate GREML models were fitted to test each Gulf War deployment exposure (ground combat exposure; insect bait exposure; oil well fire smoke exposure; pyridostigmine bromide pill use; biological/chemical warfare agent exposure; pesticide skin exposure; and pesticide uniform exposure), estimating the proportion of phenotypic variance attributable to G×E interaction on the liability scale. GREML models were adjusted for age, sex, and top 10 within-EUR genetic principal components (PC). Subsequently, we conducted single-variant G×E interaction analyses using the GEM (Gene-Environment interaction analysis for Millions of samples) software [33] and controlling for age, sex, and the top 10 PCs. Single variant G×E findings were mapped to genes using FUMA SNP2GENE function (version 1.5.0) [34]. Independent effects were defined considering linkage disequilibrium (LD) r^2^ < 0.1. To minimize potential misclassification in the G×E interaction analyses, participants who answered “not sure” were excluded from the G×E analyses.

#### PRS×E interaction

We conducted PRS×E analysis to test whether PRS previously associated with GWI [16] interact with GW deployment exposures to influence GWI risk. We calculated PRS in the GW Veterans using large-scale independent genome-wide association statistics generated from cohorts not including MVP cohort (Table S4). We used the PRS-continuous shrinkage (PRS-CS) method to infer posterior variant-level effect sizes using a high-dimensional Bayesian regression framework [35]. EUR populations available from 1000 Genomes Project Phase 3 were used as the LD reference panel. Logistic regression was performed to test PRS-GWI association and its interaction with GW deployment exposures, including age, sex, and top 10 PCs as covariates.

#### Gene ontology enrichment and drug repurposing

To investigate the biological mechanisms underlying PRS associations, we performed PRSet analysis using PRSice-2 [36, 37]. This analysis tested gene ontology (GO) terms available from the Molecular Signatures Database (MSigDB) [38]. PRSet analysis was limited to PRS showing significant interactions with GW deployment exposures. PRS were calculated by clumping full genome-wide information considering a 250 kb window and LD r^2^ = 0.1. To reduce redundancy among GO terms, we used REVIGO [39], applying a uniqueness score > 0.7, UniProt as the reference database, and SimRel semantic similarity metric. Then, we conducted an exploratory computational drug repurposing analysis using the Gene2drug approach [40], which mapped significant, non-redundant GO terms to small-molecule compounds available from the Connectivity Map [41].

#### Epigenetic association and interaction

We conducted an EWAS using the meffil pipeline [42], modeling M-values as the dependent variable. Beta values were normalized using functional normalization method [42]. No substantial batch effects in the beta values across arrays or processing batches were observed in the DNA methylation data [43]. Cell type composition was estimated using the Houseman algorithm [44] with reference data from DNA methylation profiles [45–48]. M-values were calculated from the normalized beta values for statistical analysis. The primary outcome was CDC-defined severe GWI, and models were adjusted for age, sex, top five PCs, epigenetically inferred smoking score, and estimated proportions of CD8 + T cells, CD4 + T cells, natural killer (NK) cells, B cells, and monocytes. Smoking score was inferred based on 39 CpG probes as previously described (https://github.com/PGC-PTSD-EWAS/EPIC_QC/blob/main/PGCpipeline-3.2-smoking.R). To account for bias and inflation in test statistics, we applied genomic control correction using the Bacon method [49]. We then conduct DNA methylation-environment Interaction analysis to understand how genetic and environmental factors jointly influence GWI susceptibility through epigenetic modifications. CpG sites with suggestive evidence of GWI associations (*p* < 0.01) were further tested with respect to DNA methylation interactive effects with respect to military exposure. FDR multiple testing correction (FDR q < 0.01) was applied to this follow-up analysis.

## Results

### Genome-wide G×E interaction

**Fig. 1:**
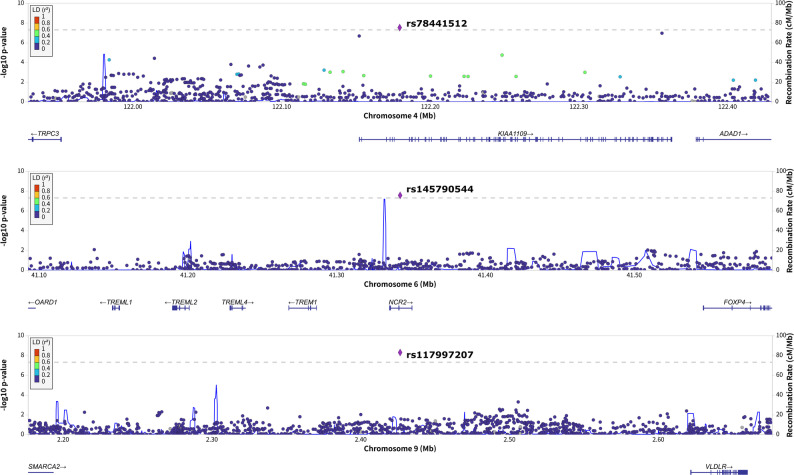
Genome-wide significant gene-environment interaction of rs78441512 (**A**), rs145790544 (**B**), and rs117997207 (**C**) with exposure to biological/chemical warfare agents in relation to their association with Gulf War Illness (GWI)

We observed nominal evidence of a genome-wide gene–environment interaction variance component (0.38 ± 0.22 on the liability scale, *p* = 0.04) related to biological/chemical warfare-agent exposure (Table S5). Because this GW exposure showed the strongest effect on GWI in a previous MVP study [14], we conducted a genome-wide G × E analysis to identify specific genetic variants showing GWI G × E interactions related to biological/chemical warfare agents. Negligible inflation was observed for both joint and interaction statistics (λ = 1.04 and 1.02, respectively). Considering loci reaching genome-wide significance (*p* < 5 × 10^− 8^) for both joint and interaction GEM statistics related to the exposure to biological/chemical warfare agents, we found three LD-independent variants (Fig. 1; Table S6): rs78441512*A (G×E beta = 1.78, joint-*p* = 8.67 × 10^− 10^, interaction-*p* = 2.95×10^− 8^), rs145790544*T (G×E beta = -2.05, joint-*p* = 1.41 × 10^− 8^, interaction-*p* = 2.75 × 10^− 8^), and rs117997207*T (G × E beta = 2.32, joint-*p* = 4.53×10^− 10^, interaction-*p* = 5.05 × 10^− 9^). With respect to loci identified by GWI candidate gene studies [50–52] and the previous GWI genome-wide association study (GWAS) (Table S7) [16], we observed nominally significant interaction with exposure to biological/chemical or warfare agents for GWAS-identified locus rs142258944*C (G×E beta = 0.625, joint-*p* = 6.04 × 10^− 10^, interaction-*p* = 0.005) and candidate locus *PON1* rs662*C (G×E beta = 0.345, joint-*p* = 0.036, interaction-*p* = 0.012).

### PRS×E interaction

**Fig. 2:**
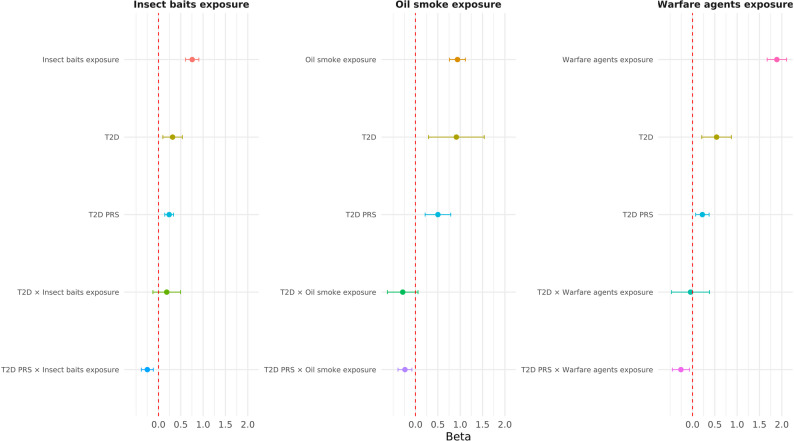
Interplay between type-2 diabetes (T2D) and Gulf War (GW) deployment exposures. Association and interactive effects of T2D polygenic risk score (PRS), T2D status, and deployment-related exposures (i.e., use of insect baits/no-pest strips in living area, close proximity to smoke from oil well fires, and exposure to biological/chemical warfare agents) with GWI. Effect size estimates with corresponding 95% confidence intervals are shown for each locus

To gain additional insight into GWI interplay between genetic and environmental factors, we tested the interaction of 12 GWI-associated PRS [16] and GW deployment exposures (Tables S4 and S8). Applying a Bonferroni correction accounting for the number of PRS tested (*p* < 0.007), T2D PRS showed significant GWI-related interactive effects with use of insect baits or no-pest strips (PRS-beta = 0.27, *p* = 2.92 × 10^− 8^; interaction-beta = -0.22, *p* = 6.7 × 10^− 4^), close proximity to smoke from oil well fires (PRS-beta = 0.34, *p* = 4.97 × 10^− 7^; interaction-beta=-0.27, *p* = 3.02 × 10^− 4^), and exposure to biological/chemical warfare agents (PRS-beta = 0.28, *p* = 1 × 10^− 4^; interaction-beta = -0.27, *p* = 0.003). The negative interactive effect indicates an attenuation of the PRS effect in GW Veterans exposed to deployment-related risk factors. To further investigate this interplay, we tested both T2D diagnosis and T2D PRS with respect to GWI and deployment exposures. When included in the same model, both T2D diagnosis and T2D PRS were independently associated with increased GWI (Table S9). However, T2D diagnosis showed a nominally significant interaction only with close proximity to smoke from oil well fires (interaction-beta=-0.42, *p* = 0.011; Table S9). When testing both main and interactive effects of T2D diagnosis and T2D PRS in the same model, the main effects remained significant for both, while significant interactive effects were only observed with respect to T2D PRS (Fig. 2; Table S9).

### Gene ontology enrichment and drug repurposing

While no GO term survived multiple testing correction, we identified 169 nominally significant, non-redundant pathway-based PRS associations (Table S10). Top GO terms included “enone reductase activity” (*p* = 5.34 × 10^− 5^), “execution phase of apoptosis” (*p* = 3.28 × 10⁻⁴), and “double-strand break repair via single-strand annealing” (*p* = 9.89 × 10⁻⁴). An exploratory computational drug repurposing analysis based on these GO terms related to GWI-T2D PRS association identified three candidate drugs surviving FDR multiple testing correction (FDR q < 0.1; Table S11): clebopride (*p* = 2.77 × 10^− 4^), rifampicin (*p* = 2.30 × 10^− 3^), and fisetin (*p* = 2.41 × 10^− 3^).

### Epigenetic association and interaction

**Fig. 3:**
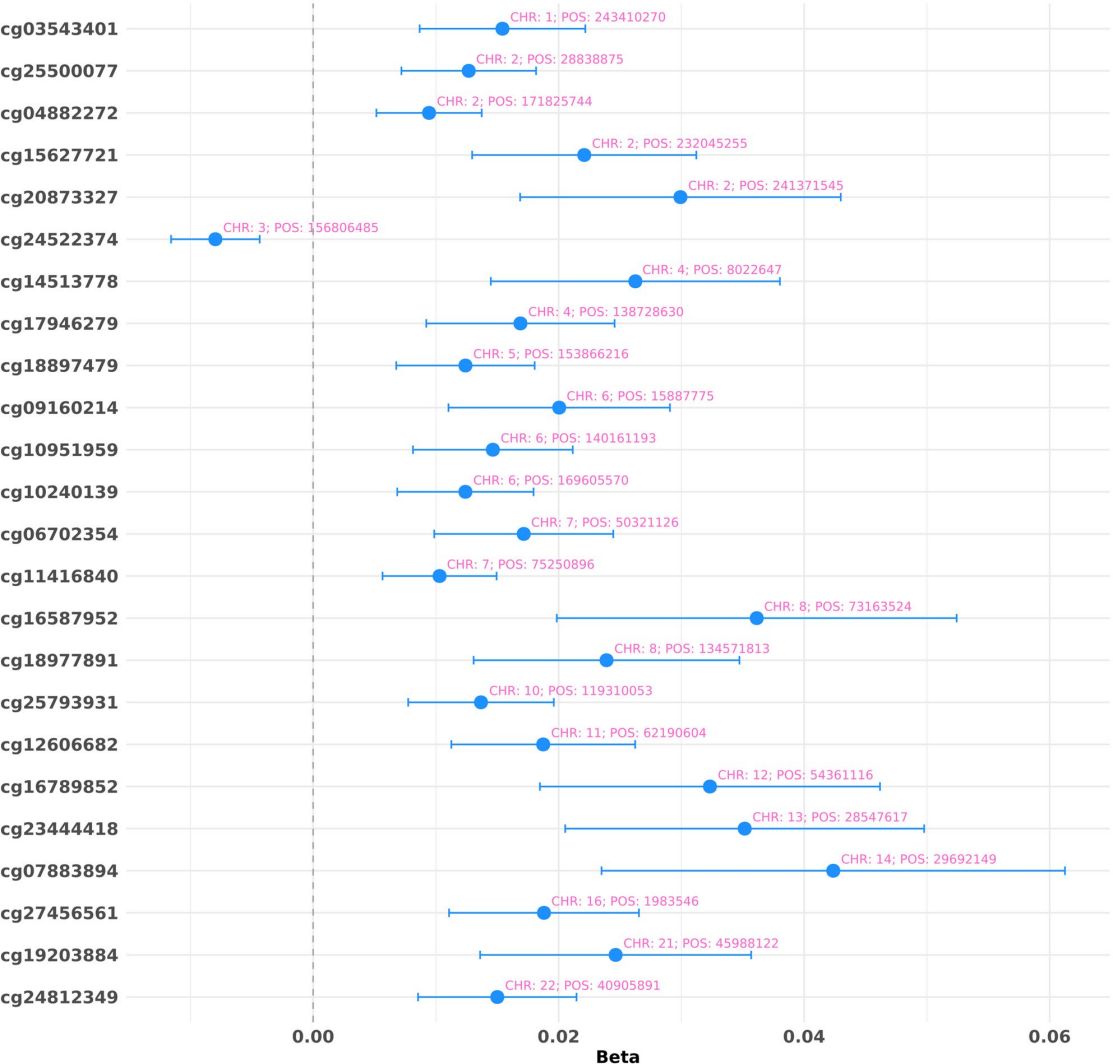
Suggestive CpG sites associated with GWI. Effect size estimates with corresponding 95% confidence intervals are shown for each CpG site

Leveraging DNA methylation data available for the GW deployed Veterans (*N* = 435), we conducted an EWAS of GWI. Due to the limited sample size, no CpG site was associated with GWI after FDR multiple testing correction. Applying a suggestive significance to explore the possible interplay between DNA methylation and GWI, we identified 24 CpG sites with suggestive evidence of association with GWI (*p* < 0.01; Table S12). Interestingly, 23 of them showed that GWI was associated with increased DNA methylation (Fig. 3), while the only inverse association was observed for cg24522374 on chromosome 3 (beta = -0.008, *p* = 9.64 × 10^− 3^). The most statistically significant epigenetic association was observed with respect to cg12606682 on chromosome 11 (beta = 0.019, *p* = 3.43 × 10^− 3^). Considering these suggestive CpG sites, we investigated epigenetic interaction with GW deployment exposures (Table S13). Applying FDR multiple testing correction to this follow-up analysis, we observed an FDR-significant interaction between cg09160214 methylation and GW ground combat (Z_interaction_ = -3.04, *p* = 9.64 × 10^− 3^, FDR q = 0.057) in the context of GWI. Among nominally significant results (*p* < 0.05), cg24522374 showed GWI epigenetic interactions with biological/chemical warfare agent exposure (Z_interaction_ = 2.137, *p* = 0.033) and having used pesticides on the skin during GW (Z_interaction_ = 2.02, *p* = 0.043). Similarly, cg23444418 showed nominally significant interaction with having used pesticides on the skin (Z_interaction_ = -1.99, *p* = 0.047) and pyridostigmine bromide pills during GW (Z_interaction_ = -2.46, *p* = 0.014).

In line with the previously observed GWI-T2D genetic overlap [16], two of the CpG sites suggestively linked with GWI were previously identified as associated with T2D [53]: cg15627721 (GWI beta = 0.022, *p* = 4.63 × 10^− 3^; T2D beta = 0.081, *p* = 9.2 × 10^− 5^) and cg06702354 (GWI beta = 0.017, *p* = 4.85 × 10^− 3^; T2D beta = 0.073, *p* = 2.2 × 10^− 5^). However, these CpG sites did not show significant interactions with GW deployment exposures (*p* > 0.05).

## Discussion

Investigating 1990–1991 GW Veterans enrolled in the MVP cohort, we assessed genetic and epigenetic interplay between GW-deployment exposures and GWI. Our findings highlighted the role of G×E interactions in the etiology of GWI. In particular, we observed nominal evidence of a genome-wide gene–environment interaction variance component (0.38 ± 0.22 on the liability scale) related to exposure to biological/chemical warfare agents. While this is in line with multiple studies highlighting the impact of this deployment risk factor on GWI pathogenesis [54, 55], the degree of uncertainty of the estimate observed strongly indicate the need of larger sample size to follow up on this initial finding.

Our single-variant G×E analysis identified three variants with genome-wide significant evidence of G×E interactions related to exposure to chemical or biological warfare agents. Interestingly, these variants had MAF < 10%. We did not observe major inflation in the genome-wide interaction statistics of variants with MAF < 10% (λ = 1.07). There is a well-known inverse relationship between allele frequency and effect size due to the effect of background selection [56, 57]. Accordingly, the larger effect sizes may have increased the statistical power of our analyses to detect G×E interactions in low-allele frequency variants. Rs78441512 was located in *BLTP1* (formerly known as *KIAA1109*), a gene implicated in various cellular processes such as lipid transport and membrane dynamics [58]. Based on GTEx v10 data [59], this variant regulates *BLTP1* expression in 19 tissues (details available at https://www.gtexportal.org/home/snp/rs78441512). Other *BLTP1* variants identified by previous GWAS have been associated with eosinophil percentage [60], celiac disease [61], eczema [62], asthma [63], depression [64], and inflammatory bowel disease [65], suggesting a potential role in immune regulation and systemic inflammation. This supports a mechanistic link between *BLTP1* variation and the immune dysregulation observed in GWI. Rs145790544 mapped to *NCR2*, a gene encoding a triggering receptor on NK cells [66]. Considering GTEx v10 data [59], this variant is a blood eQTL for *TREML4* and three pseudogenes (i.e., *TREML5P*, *ENSG00000278745*, and *ENSG00000274256*; https://www.gtexportal.org/home/snp/rs145790544). *TREML4* encodes a protein expressed in dendritic cells and macrophages and plays a role in antigen presentation and inflammatory responses [67]. The fact that chromosome-4 and chromosome-6 loci are implicated in immune function supports the hypothesis that toxicant-triggered immune dysregulation may be central to GWI pathogenesis [55, 68]. The other G×E interaction was related to rs117997207, which mapped to *VLDLR-AS1*, a long non-coding RNA previously correlated with combat-related chronic mild traumatic brain injury and depression symptoms in US Veterans [69]. Other *VLDLR-AS1* variants have been identified by previous GWAS as associated with hemoglobin concentration, hematocrit, platelet count, and neutrophil count [60, 70], suggesting a potential connection to neurovascular and immune pathways. Together, these findings implicate genes involved in immune regulation, inflammatory responses, and cellular trafficking as potentially implicated in the interplay between military exposures and GWI. While the precise molecular mechanisms remain to be elucidated, our findings provide biologically plausible pathways for further functional studies.

With respect to loci previously linked to GWI (Table S7), we observed nominally significant G×E interactions related to exposure to chemical or biological warfare agents for *BMPR1B* rs142258944 and *PON1* rs662. The first was identified by a previous GWI GWAS conducted in the MVP cohort [16]. *BMPR1B* gene has been implicated in the regulation of bone morphogenesis and reproductive function [71], but there is limited information regarding its potential relationship with GWI or with vulnerability to deployment-related exposures. Conversely, previous studies linked *PON1* rs662 to GWI, because of its impact on altering the detoxification activity of paraoxonase-1 enzyme [50–52]. However, concerns were raised that population stratification biases may have contributed to some of *PON1* rs662 findings [72]. In the present study, after controlling for the genetic structure of the study population, we observed *PON1* rs662 G×E interaction for GWI but this effect was only nominally significant.

Overall, the effect of the individual variants explains only a small portion of the overall variance potentially accounted by G×E interaction. Accordingly, we hypothesize that there is no single locus responsible for the Veterans’ overall vulnerability to GW deployment exposures, but rather multiple genes contribute to the interplay between military risk factors and GWI. In this context, our PRS analyses permitted us to investigate polygenic mechanisms interacting with GW deployment exposures. Specifically, we observed that T2D PRS interacted with chemical or biological warfare agents’ exposure, insect baits/no-pest strips exposure, and close proximity to smoke from oil well fires. Similar to chemical or biological warfare agents [54, 55], pesticides and oil well smoke have been linked to GWI in deployed Veterans [14]. Increased diabetes prevalence has also been reported in Veterans with GWI and also in those reporting toxican exposure [73, 74]. Additionally, a GWI mouse model exposed to GW-related chemicals (including pesticides) developed transient insulin insensitivity along with fatigue and learning deficits [75]. In our study, while T2D PRS and T2D status were independently associated with GWI, interactive effects with respect to GW deployment exposures were observed only with respect to T2D PRS. This suggests that T2D PRS relationship with GWI is independent of its effect on T2D risk, perhaps due to pathogenic processes shared between GWI and T2D. Previous research highlighted specific metabolic features of GWI, also including alterations in lipid and purine metabolism [76]. These metabolic disruptions could provide a plausible biological context for the relationship among T2D PRS, GW exposures, and GWI. Importantly, further studies will be needed to understand the dynamics underlying the attenuation of the T2D PRS effect in GW Veterans exposed to deployment-related risk factors. Multiple scenarios may be involved, such as the competing effect of T2D PRS and GW deployment on GWI pathogenesis or gene-environment correlations related to the potential effect of T2D on the characteristics of the military personnel enrolled and deployed during GW.

Although GO terms did not survive multiple testing correction, our computational drug-repurposing analysis based on GWI-T2D PRS association identified three FDR-significant compounds: clebopride, rifampicin, and fisetin. Clebopride is a D2 dopamine receptor antagonist that acts on the central nervous system to suppress vomiting and treat dyspepsia (details available at https://go.drugbank.com/drugs/DB13511). Rifampicin (also known as rifampin) is an antibiotic used to treat mycobacterial infections (e.g., tuberculosis) and gram-positive bacterial infections (details available at https://go.drugbank.com/drugs/DB01045). Of note, rifampicin has also shown some evidence of neuroprotective effect [77]. Fisetin is a dietary antioxidant reported to have neurotrophic, anticarcinogenic, anti-inflammatory, and other health beneficial effects [78]. These compounds highlight how genetic information can be used to identify potential targets to develop therapeutic interventions for GWI. While these results are potentially promising, it is important to emphasize the exploratory character and the computational design of this analysis. Accordingly, the identification of these compounds does not imply therapeutic efficacy or clinical applicability at this stage. Instead, these findings illustrate how genetic and pathway-level information can generate hypothesis-driven candidates for further experimental validation.

Our exploratory GWI EWAS identified several suggestive epigenetic associations with GWI. The strongest statistical evidence was observed with respect to cg12606682, a CpG located in the 3’UTR of *SCGB1A1* gene, which is known for its role in pulmonary and immune function [79]. While 23 out of 24 CpG sites showed increased methylation associated with GWI, cg24522374 presented an inverse relationship (i.e., higher methylation was associated with lower GWI odds). Notably, cg24522374 also showed nominal interactions with warfare agent exposure and having used pesticides on the skin during GW. This CpG site is located within a promoter region near a CpG island (3:156806178–156807773). GWI-related reduced DNA methylation at this promoter and the interactive effects observed may alter its activity and consequently alter transcription regulation of the related genes. We also identified FDR-significant interaction between cg09160214 and exposure to GW ground combat. This CpG site is located within a DNase hypersensitivity region (PsychENCODE chr6:15887680–15887915). Altered methylation in DNase hypersensitivity sites can modify the binding of regulatory proteins, influencing transcription regulation [80]. Finally, cg23444418 also showed suggestive evidence of interaction with both having used pesticides on the skin and pyridostigmine bromide pills during GW. This CpG site is located upstream of a CpG island (chr13:28549839–28550246). Similarly to the other loci, altered DNA methylation in this regulatory element may alter transcriptomic regulation, contributing to GWI pathogenesis [81].

Interestingly, CpG sites potentially associated with GWI that did not show interactions with GW deployment exposures were located near or within gene body regions. In line with GWI multisystemic nature, the loci identified appear to be related to diverse biological processes including cytoskeletal organization (*CEP170* cg03543401) [82], glycosylation (*ST3GAL1* cg18977891) [83], neuronal network remodeling (*MKL1* cg24812349) [84], neuronal development (*ABLIM2* cg14513778) [85], lipid metabolism (*PLB1* cg25500077) [86], and intracellular trafficking (*HIP1* cg11416840) [87]. Additionally, two of the CpG sites identified in our GWI EWAS (cg15627721 and cg06702354) were previously associated with T2D [53]. Both cg15627721 on chromosome 2 and cg06702354 on chromosome 7 are located within open chromatin (chr2:232044494–232045366; chr7:50320372–50323392) and transcription binding regions (chr2:232044508–232045328; chr7:50320227–50324475). Cg15627721 is located near *PSMD1* and *ARMC9* genes, which are implicated in cellular apoptosis and protein transport [88, 89]. This appears to converge with T2D PRS results, which identified “execution phase of apoptosis” as one of the top GO enrichments. Cg06702354 is located near *IKZF1*, which encodes a transcription factor essential for chromatin remodeling and lymphocyte differentiation regulation [90].

Our study has several limitations. First, although our cohort is the largest available to study GWI to date, the sample size remains insufficient to adequately investigate GWI polygenic architecture and its interplay with GW deployment-related exposures. In this context, we had to limit our analyses to the EUR sample, because of the smaller sample size of the other population groups available among MVP-GW Veterans (*N* < 2750). Similarly, we could not perform a multivariable GREML analysis modeling multiple exposures simultaneously. Instead, to limit the burden of multiple testing correction, we had to limit our single-variant G×E analysis to the exposure to biological/chemical warfare agents, as this was the only nominally significant GW exposure in the GREML analysis. While this GW exposure showed the strongest effect among those assessed in the MVP cohort [14], G×E loci are also expected to be present with respect to the other GW-deployment exposures. A larger sample size will be needed to adequately explore G×E analysis across GW deployment-related exposures and population groups. Additionally, because an underpowered genome-wide investigation can be affected by the winner’s curse [91], our G×E findings will need to be confirmed in more statistically powerful samples. Similarly, larger cohorts will be required to follow up our exploratory GWI EWAS. Another important limitation is related to the assessment of GW exposures. While participants who responded “not sure” for a given exposure were excluded to minimize potential misclassification in the G×E analyses, the retrospective and self-reported nature of environmental exposure data introduces potential recall biases. Accordingly, subjective exposure assessment used in the present may have reduce measurement precision and weaken gene-environment interaction signals [92]. However, this bias is expected to have reduced our statistical power rather than to have induced false positive results. Finally, the cross-sectional design of the study limits causal inference, which is a common issue in environmental health research fields [93]. While we identified gene-environment interactive effects and epigenetic interactions, the temporal relationship between environmental exposures, molecular changes, and GWI clinical onset requires further investigation.

## Supplementary Information

Below is the link to the electronic supplementary material.

Supplementary Material 1.

Supplementary Material 2.

## Data Availability

All data generated in this study are provided within the manuscript and its supplementary materials. In accordance with the Million Veteran Program’s data sharing policy, omic-wide association summary statistics will be made publicly available through the Database of Genotypes and Phenotypes (dbGaP; Study Accession: phs001672). Due to the policies of the Department of Veterans Affairs and the informed consent signed by Million Veteran Program participants, we are not allowed to share individual-level data.

## Code availability

This study did not involve the reporting of novel or original code. The code used in the current study is available at https://yanglab.westlake.edu.cn/software/gcta/#GREML, https://github.com/large-scale-gxe-methods/GEM, https://github.com/getian107/PRScs, https://choishingwan.github.io/PRSice/prset_detail/, https://github.com/PGC-PTSD-EWAS/EPIC_QC, and https://github.com/perishky/meffil.
